# The peroxisome proliferator activated receptor gamma agonist pioglitazone increases functional expression of the glutamate transporter excitatory amino acid transporter 2 (EAAT2) in human glioblastoma cells

**DOI:** 10.18632/oncotarget.4019

**Published:** 2015-06-03

**Authors:** Jared Ching, Stephanie Amiridis, Stanley S. Stylli, Andrew R. Bjorksten, Nicole Kountouri, Thomas Zheng, Lucy Paradiso, Rodney B. Luwor, Andrew P. Morokoff, Terence J. O'Brien, Andrew H. Kaye

**Affiliations:** ^1^ Department of Surgery, The University of Melbourne, The Royal Melbourne Hospital, Victoria, Australia; ^2^ Department of Medicine, The University of Melbourne, The Royal Melbourne Hospital, Victoria, Australia; ^3^ Department of Anaesthesia and Pain Management, The Royal Melbourne Hospital, Victoria, Australia; ^4^ Department of Neurosurgery, The Royal Melbourne Hospital, Victoria, Australia

**Keywords:** glutamate, pioglitazone, PPAR gamma, glioblastoma multiforme, EAAT2

## Abstract

Glioma cells release glutamate through expression of system x_c_^−^, which exchanges intracellular glutamate for extracellular cysteine. Lack of the excitatory amino acid transporter 2 (EAAT2) expression maintains high extracellular glutamate levels in the glioma microenvironment, causing excitotoxicity to surrounding parenchyma. Not only does this contribute to the survival and proliferation of glioma cells, but is involved in the pathophysiology of tumour-associated epilepsy (TAE). We investigated the role of the peroxisome proliferator activated receptor gamma (PPARγ) agonist pioglitazone in modulating EAAT2 expression in glioma cells. We found that EAAT2 expression was increased in a dose dependent manner in both U87MG and U251MG glioma cells. Extracellular glutamate levels were reduced with the addition of pioglitazone, where statistical significance was reached in both U87MG and U251MG cells at a concentration of ≥ 30 μM pioglitazone (*p* < 0.05). The PPARγ antagonist GW9662 inhibited the effect of pioglitazone on extracellular glutamate levels, indicating PPARγ dependence. In addition, pioglitazone significantly reduced cell viability of U87MG and U251MG cells at ≥ 30 μM and 100 μM (*p* < 0.05) respectively. GW9662 also significantly reduced viability of U87MG and U251MG cells with 10 μM and 30 μM (*p* < 0.05) respectively. The effect on viability was partially dependent on PPARγ activation in U87MG cells but not U251MG cells, whereby PPARγ blockade with GW9662 had a synergistic effect. We conclude that PPARγ agonists may be therapeutically beneficial in the treatment of gliomas and furthermore suggest a novel role for these agents in the treatment of tumour associated seizures through the reduction in extracellular glutamate.

## INTRODUCTION

Glutamate is the most abundant neurotransmitter in the mammalian central nervous system and mediates its excitatory physiological effect by binding to ionotropic and metabotropic receptors. [1, 2] Glioma cells release glutamate through expression of the x_c_^−^ exchanger, which exchanges intracellular glutamate for extracellular cysteine. [3] Intracellular uptake of cysteine via system x_c_^−^ permits glioma cell survival through glutathione formation. Excitatory amino acid transporter 2 (EAAT2) is one of 5 subtypes of sodium dependent plasma membrane glutamate transporters that accounts for up to 90% of extracellular glutamate uptake, with Glutamate Aspartate Transporter (GLAST-1) accounting for the largest remaining proportion. [4, 5] Glioma cell lines lack expression of both EAAT2 and GLAST-1, which is associated with impaired glutamate uptake. [3, 6] Together, these features result in abnormally high extracellular glutamate concentrations resulting in excitotoxicity, causing necrosis of the adjacent parenchyma, which creates space permitting proliferation. [7, 8] Additionally, cysteine uptake provides a precursor for the formation of glutathione, which is protective against endogenous reactive oxygen species and further promotes glioma growth. [7] Furthermore, deranged glutamate transport has been associated with the pathogenesis of glial tumour associated epilepsy (TAE). [9, 10]

PPARγ is a ligand-dependent transcription factor that responds to both physiological and chemical stimuli, including the cyclopentatone prostaglandin, 15-deoxyΔ^12, 14^ prostaglandin J_2_ (15d-PGJ_2_) and thiazolidinediones (TDZ) respectively. [11] Expression of PPARγ in the brain has been found in multiple cell types including microglia, astrocytes, oligodendrocytes, and neurons. It has been shown in rat cortical cultures incubated with the commercially available PPARγ agonist rosiglitazone, that this drug increases the expression levels of EAAT2 at both the protein and mRNA levels. [12] Furthermore, reduction in the infarct volume after administration of rosiglitazone in rats with middle cerebral artery occlusion demonstrated in this study strongly supports the clinical evidence of better neurological outcomes found in a small case-matched controlled study investigating stroke recovery with TDZ drugs. [12, 13]

Increased PPARγ expression has been associated with beneficial effects in cancers including breast and colon, including a reduction in cell proliferation and improved patient prognoses. [14–17] In comparison to other cancer cell lines, it has been observed that glioma cells express lower endogenous levels of PPARγ. [18] Nevertheless, there is a growing body of evidence that demonstrates several mechanisms by which PPARγ agonists elicit anti-neoplastic effects in gliomas. Pioglitazone has been shown to reduce the expression of β-catenin independently of PPARγ. [19] Others have observed that the PPARγ agonist citaglitazone mediates glioma cell apoptosis independently of PPARγ through the reduction of Akt and induction of mitochondrial membrane potential loss. [18] Citaglitazone has also been shown to inhibit proliferation of brain tumour stem cells and expression of stemness genes in de-differentiated glioma cell lines. [20] Most recently, Grommes and colleagues demonstrated the efficacy of pioglitazone in reducing tumour growth with a human glioma xenograft model, highlighting the ability of pioglitazone to cross the blood brain barrier. [21]

Glycogen Synthase Kinase 3 (GSK3) is a serine/threonine protein kinase, which was first characterised in 1980 and is inactivated by phosphorylation at serine 21 in GSK-3α or serine 9 in GSK3-β. [22, 23] PPARγ agonists are known to be GSK3 inhibitors, which has been shown to have a neuroprotective role in stroke models through the promotion of cell survival. [24, 25] Although active GSK3 has been known to inhibit survival pathways, the inhibition of GSK3 has been paradoxically shown to be effective against several neoplasms. [26, 27] GSK3 inhibition in glioma stem cells has also been observed to induce differentiation and regulate proliferation by Korur and colleagues. [28]

As glioma cells express some PPARγ, we were interested in the potential of PPARγ agonists in increasing the expression of the glutamate transporter EAAT2. Herein, we describe a novel mechanism of the PPARγ agonist, pioglitazone, in which we demonstrate its ability to increase EAAT2 expression and consequently extracellular glutamate levels in glioma cells. In addition we show that this agent alters cellular morphology, whilst also reducing the viability of human glioblastoma cell lines. These results warrant further investigation into the potential role of this class of agents in both anti-neoplastic and anti-convulsant therapy in gliomas.

## RESULTS

### Human glioblastoma multiforme (GBM) cell lines express low levels of the glutamate transporters EAAT2 and GLAST-1

**Figure 1:**
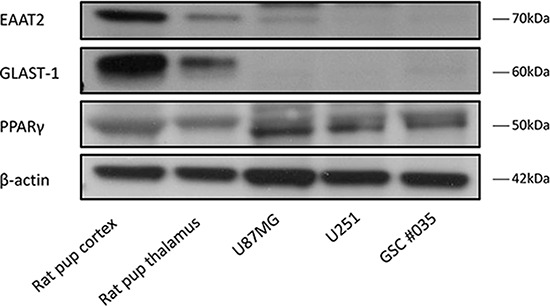
Representative blots showing EAAT2, GLAST-1, PPARγ and β-actin expression in rat cortex, thalamus and glioma cells EAAT2 and GLAST-1 are expressed in low levels in glioblastoma cell lines and glioma stem cells.

We established the baseline expression of the glutamate transporters EAAT2 and GLAST-1 (EAAT1) in a number of glioma cell lines (U87MG, U251MG and GSC #35) relative to rodent normal brain tissue as a control. EAAT2 and GLAST-1 were expressed in low levels in these cell lines in comparison to the rat brain cortex and thalamus control tissues (Figure 1). This was in keeping with previous literature results with respect to EAAT2 and GLAST-1 basal expression levels observed in U251MG and U87MG cells respectively. [6, 29] Interestingly, we detected higher basal levels of PPARγ in the glioma cells compared to the normal brain tissue controls (Figure 1).

### Pioglitazone increases expression of EAAT2 and PPARγ but not GLAST-1 in U87MG and U251MG cells

**Figure 2:**
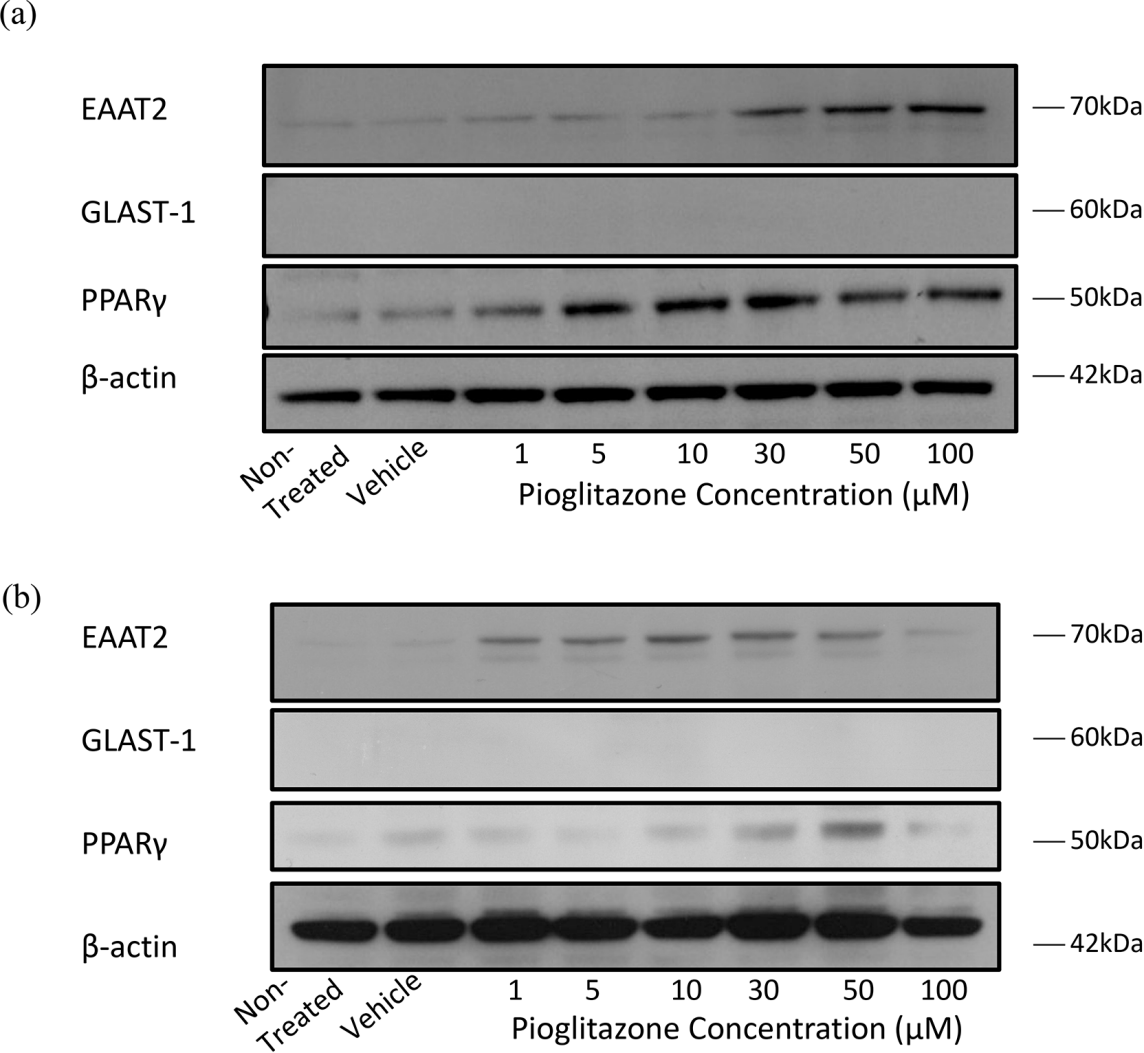
Representative blots showing EAAT2, GLAST-1, PPARγ and β-actin expression in glioma cells treated with increasing concentrations of pioglitazone for 48 hours Representative blots showing EAAT2, GLAST-1, PPARγ and β-actin expression in **(a)** U87MG and **(b)** U251MG glioma cells treated with increasing concentrations of pioglitazone for 48 hours.

**Figure 3:**
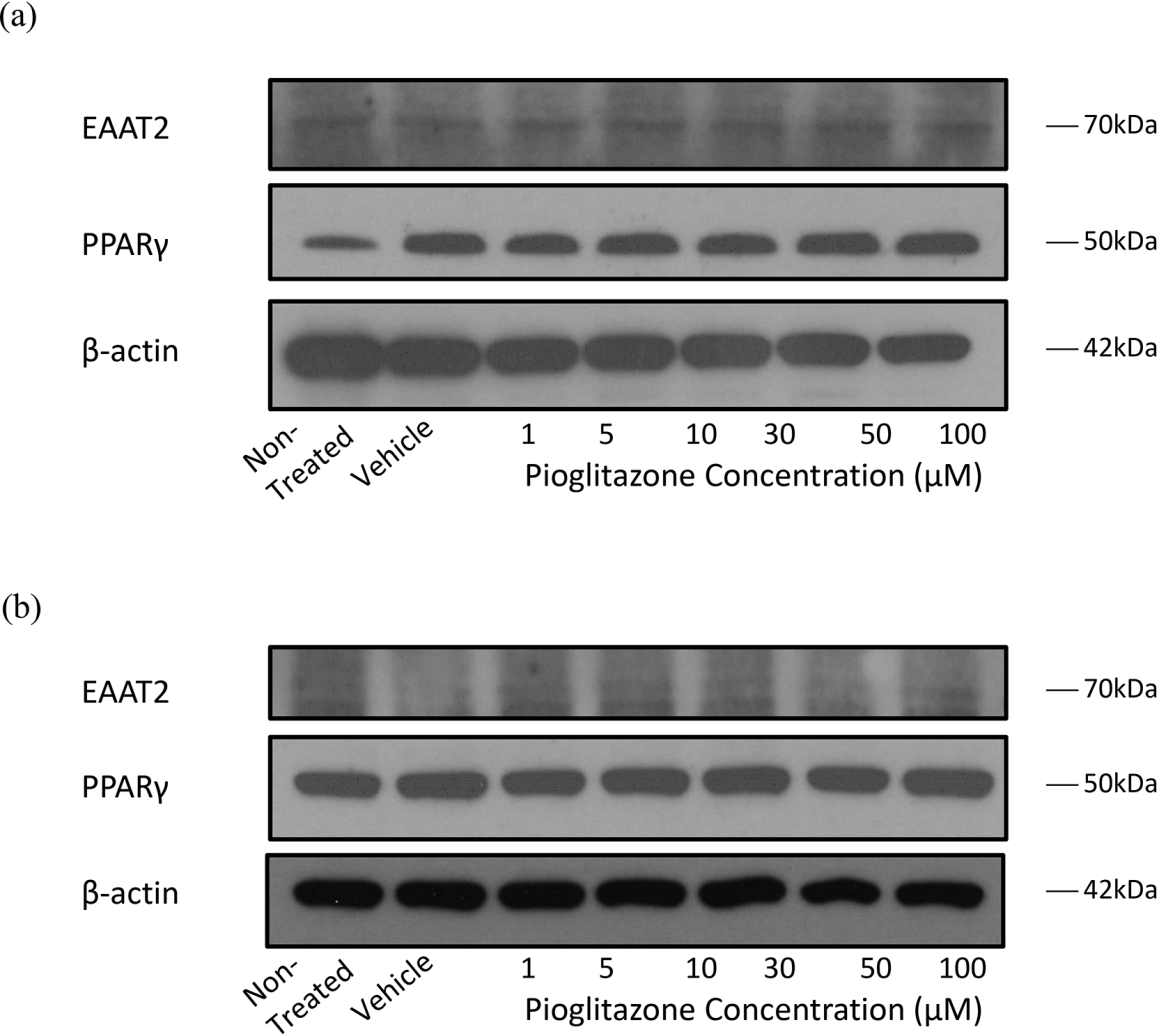
Representative blots showing EAAT2, PPARγ and β-actin expression in GSC #035 cells treated with increasing concentrations of pioglitazone for 72 hours alone (a) and with 5 μM GW9662 (b)

To determine whether glutamate transporters in glioma cells could be modulated with PPARγ agonists as with astrocytes, we treated glioma cells with increasing concentrations of pioglitazone. Incubating U87MG and U251MG cell lines with pioglitazone resulted in a dose dependent activation of EAAT2 and PPARγ but not GLAST-1 protein expression (Figure 2). PPARγ protein expression was also increased in this manner. We confirmed that EAAT2 expression is unchanged in GSC #35 by pioglitazone (Figure 3).

### Pioglitazone reduces the cell viability of GBM cell lines

**Figure 4:**
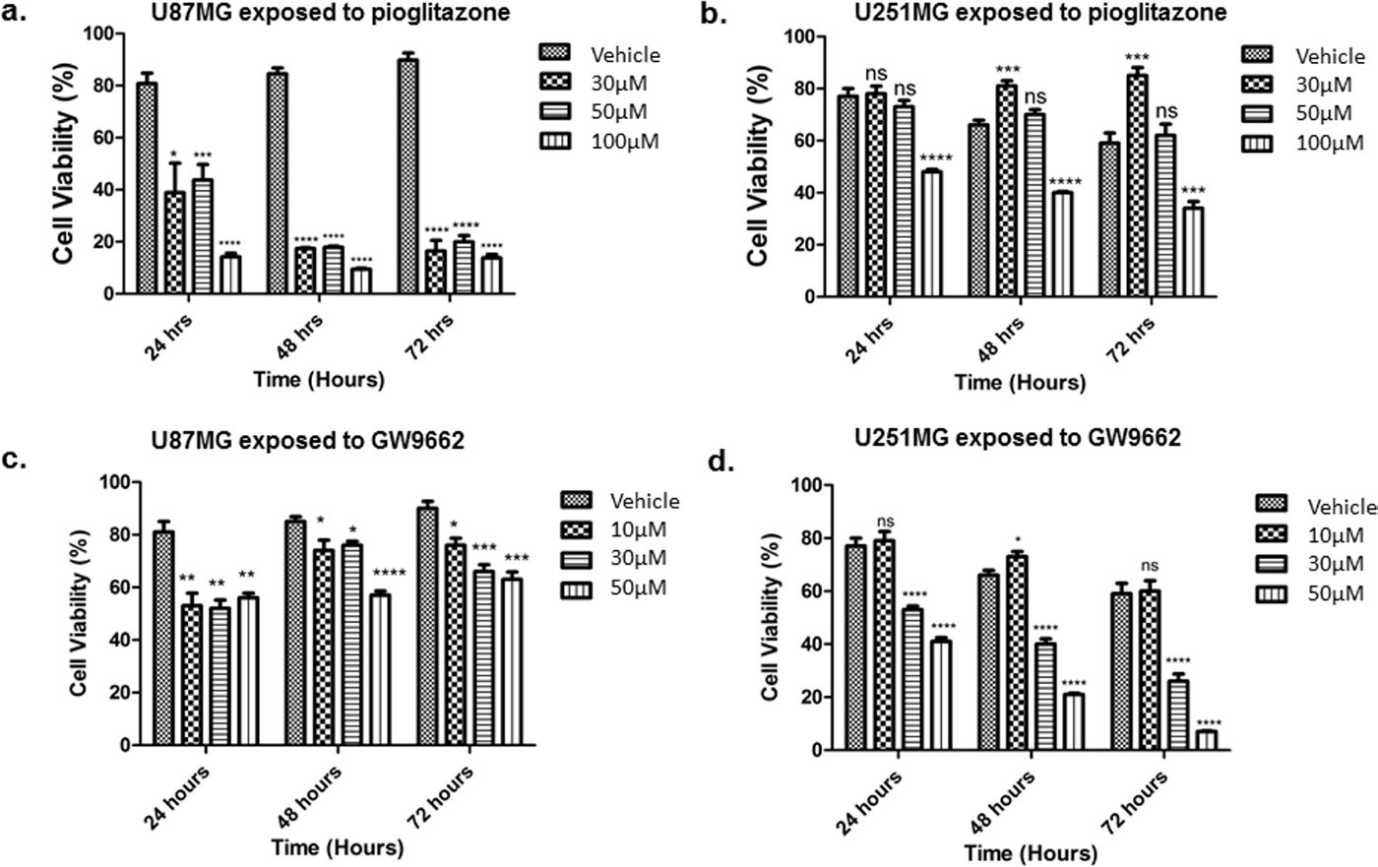
Effect of increasing concentrations of pioglitazone on U87MG (a) and U251 (b) glioma cells Pio concentrations of 30 μM U87MG cells (a) and a concentration of 100 μM in U251MG (b) results in a significant reduction in cell viabilityEffect of increasing concentrations of GW9662 on U87MG **(c)** and U251 **(d)** Cell lines. (c) GW9662 concentration of 10 μM or above resulted in a significant reduction of cell viability in U87MG glioma cells. (d) GW9662 concentration of 10 μM and above were required for a significant reduction in cell viability of U251MG cells. Bars show mean with SEM, ns refers to non-significance, and asterisks indicate statistical significance where *p = 0.01 to 0.05, **p = 0.001 to 0.01, ***p < 0.001, and ****p < 0.0001. Student's *t*-test, *n* = 4 (a), *n* = 6 (b).

Concentrations of pioglitazone of 30 μM or above resulted in a significant reduction in cell viability of U87MG cells (Figure 4(a)). In U251MG glioma cells, a significant reduction in cell viability was only observed at a pioglitazone concentration of 100 μM (Figure 4(b)). However, pioglitazone was not found to elicit cytotoxicity in GSC #35 (Figure 6(a-h)).

### GW9662 reduces viability of GBM cell lines

U87MG cell viability was reduced significantly when exposed to 10 μM or above of the PPARγ blocker GW9662 (Figure 4(c)), whereas a reduction in U251MG cell viability was observed at concentrations of 30 μM or above (Figure 4(d)). GSC #35 did not demonstrate such sensitivity to GW9662 (Figure 6(b–d)).

### Pioglitazone efficacy is partially dependent on PPARγ activation in U87MG but not in U251MG cells

**Figure 5:**
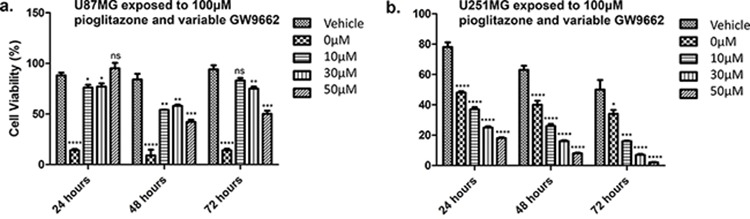
Effect of increasing concentrations of GW9662 with co-treatment of high dose (100 μM) pioglitazone on U87MG (a) and U251MG (b) glioma cell lines (a) Inhibition of PPAR with GW9662 reduced the fall in cell viability that was caused by a high concentration of pioglitazone alone(b) Pioglitazone and GW9662 co-treatment result in synergistic decrease cell viability of U251MG cells. Bars show mean with SEM, ns refers to non-significance, and asterisks indicate statistical significance where *p = 0.01 to 0.05, **p = 0.001 to 0.01, ***p < 0.001, and ****p < 0.0001. Student's *t*-test, *n* = 4 (a), *n* = 6 (b).

The loss in cell viability caused by a cytostatic concentration of pioglitazone in U87MG cells was diminished when co-incubated with GW9662 at a concentration of 10 μM or above (Figure 5(a)). Earlier we showed that GW9662 alone significantly reduced U87MG cell viability at the same concentrations, but in combination with pioglitazone there was a reduced effect compared to pioglitazone alone. U251MG cells demonstrated an opposing phenomenon whereby increasing doses of GW9662 resulted in a significant dose dependent reduction in cellular viability (Figure 5(b)).

**Figure 6:**
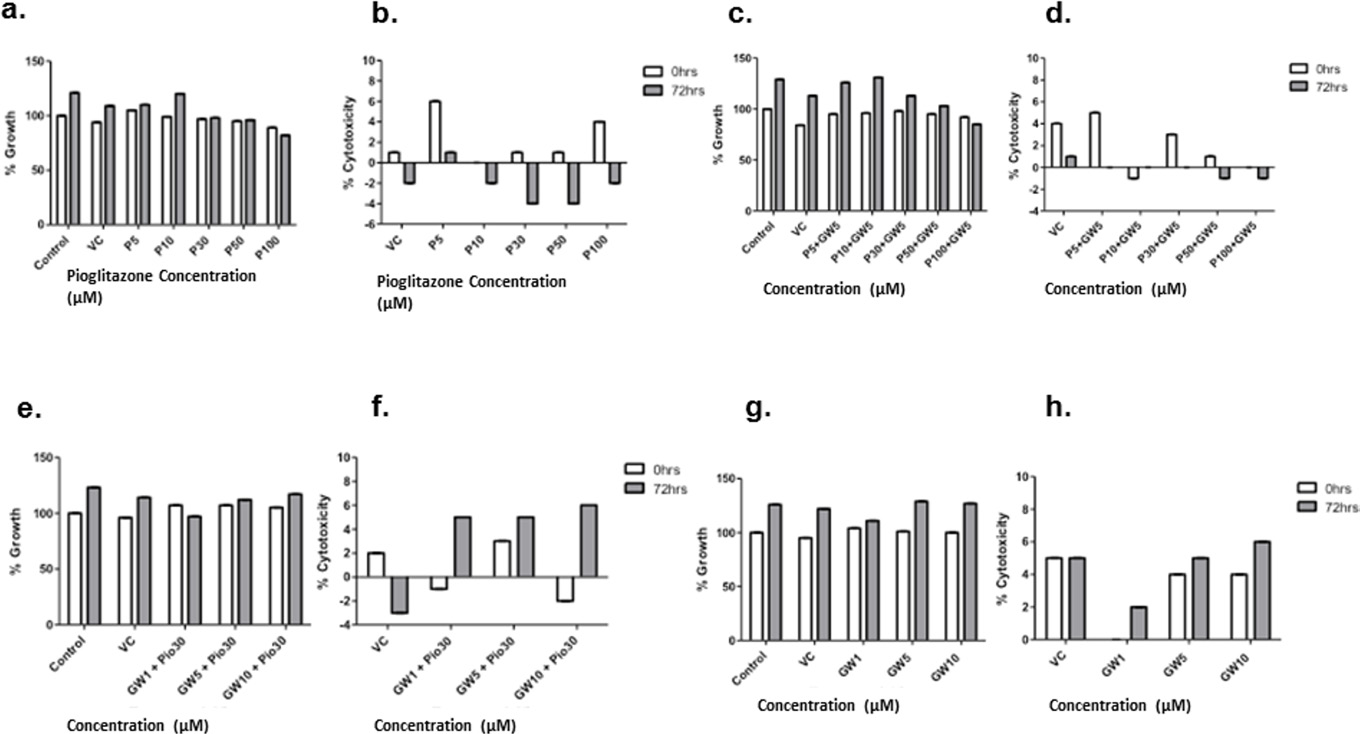
Effect of increasing concentrations of Pioglitazone and/or GW9662 treatment on glioma stem cell line #035 Cell growth and cell death was detected using the LDH assay at 0 hour and 72 hours. Addition of 30 μM or more of Pioglitazone reduces cell growthat 72 hours **(a)** however this reduction is lessened by the addition of GW9662 **(c** & **e)** GW alone does not promote cell growth compared to the control **(g)** Treatment with increasing concentrations of GW9662 and 30 μM of Pioglitazone causes reduction of growth compared to GW 9662 alone. No treatments caused significant cytotoxicity further supporting Pioglitazone as a cytostatic agent **(b, d, f** & **h)** Bars show means with SEM, ns refers to non-significance. Student *t* test, *n* = 3 (a–d). All results were non-significant, *p* value > 0.05.

### Pioglitazone reduces extracellular glutamate release from glioma cells

**Figure 7:**
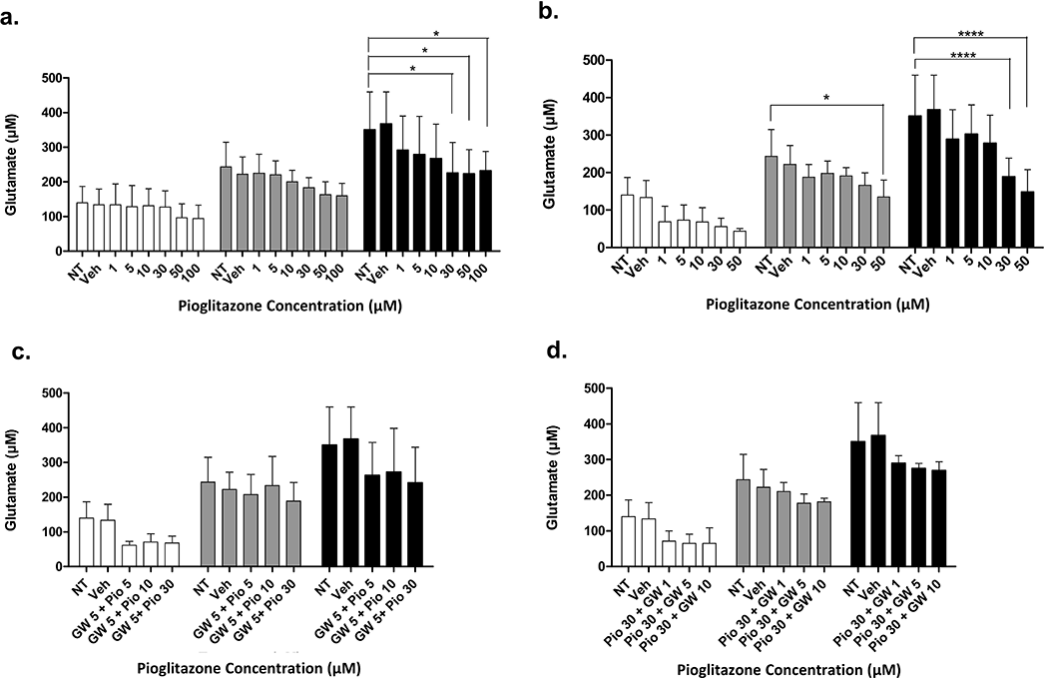
Extracellular glutamate levels measured with HPLC in U87MG cells incubated with varying concentrations of pioglitazone **(a)** GW9662 **(b)** GW9662 + pioglitazone (**c** & **d**) for 24, 48 and 72 hours (*n* = 6)A significant reduction in glutamate release was elicited at a concentration of ≥ 30 μM of pioglitazone at 72 hours (a). GW9662 at was associated with a significant reduction of glutamate at 48 and 72 hours using 50 μM and ≥ 30 μM respectively (b). There was no significant difference in glutamate release with co-administration of pioglitazone and GW9662 (c & d). Bars show mean with SEM and asterisks indicate statistical significance where *p = 0.01 to 0.05, **p = 0.001 to 0.01, ***p < 0.001, and ****p < 0.0001. ANOVA with Bonferroni Post hoc test, *n* = 6. Abbreviations: NT: non-treated, Veh: Vehicle control, Pio: Pioglitazone, GW: GW9662.

**Figure 8:**
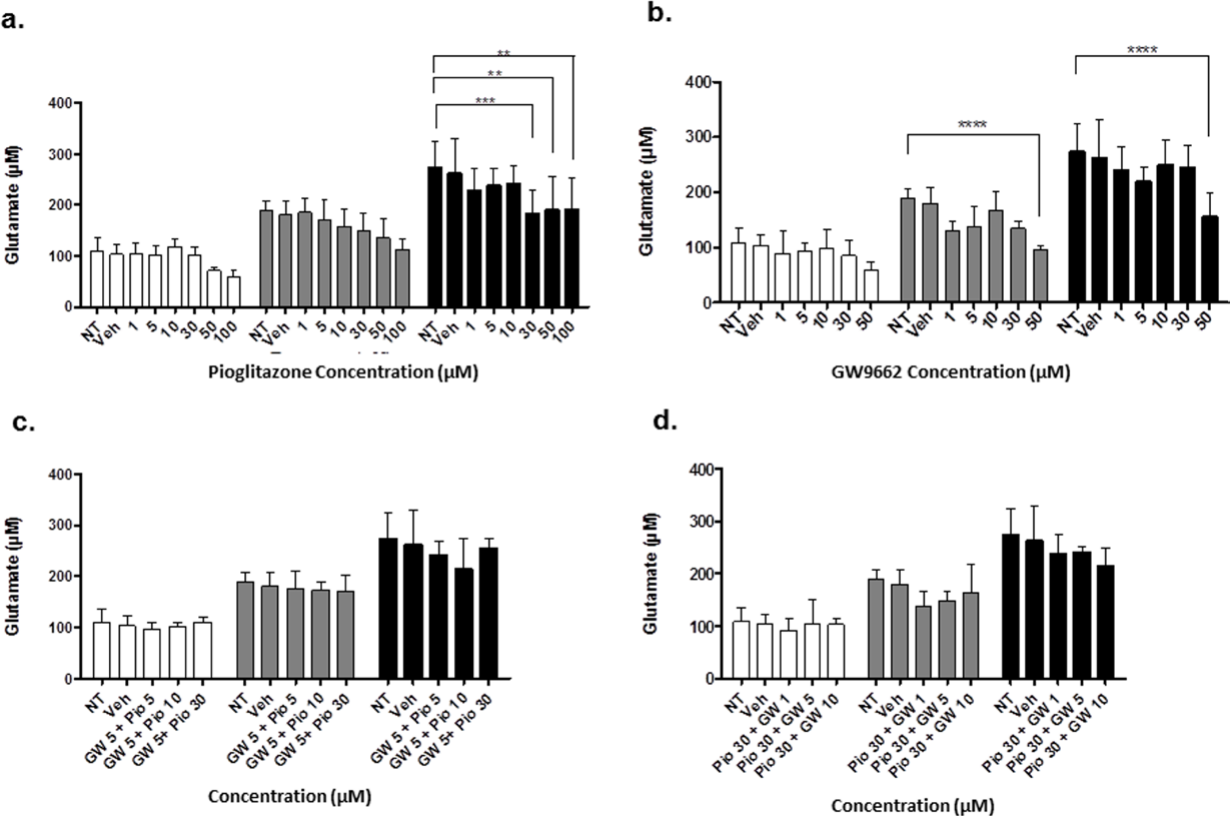
Extracellular glutamate levels measured with HPLC in U251MG cells incubated with varying concentrations of pioglitazone (a) GW9662 (b) GW9662 + pioglitazone (c & d) for 24, 48 and 72 hours (*n* = 6) A significant reduction in glutamate release was elicited at a concentration of ≥ 30 μM of pioglitazone at 72 hours (a). GW9662 at was associated with a significant reduction of glutamate at 48 and 72 hours using 50 μM (b). There was no significant difference in glutamate release with co-administration of pioglitazone and GW9662 (c & d). Bars show mean with SEM and asterisks indicate statistical significance where *p = 0.01 to 0.05, **p = 0.001 to 0.01, ***p < 0.001, and ****p < 0.0001. ANOVA with Bonferroni Post hoc test, *n* = 6. NT: non-treated, Veh: Vehicle control, Pio: Pioglitazone, GW: GW9662.

**Figure 9:**
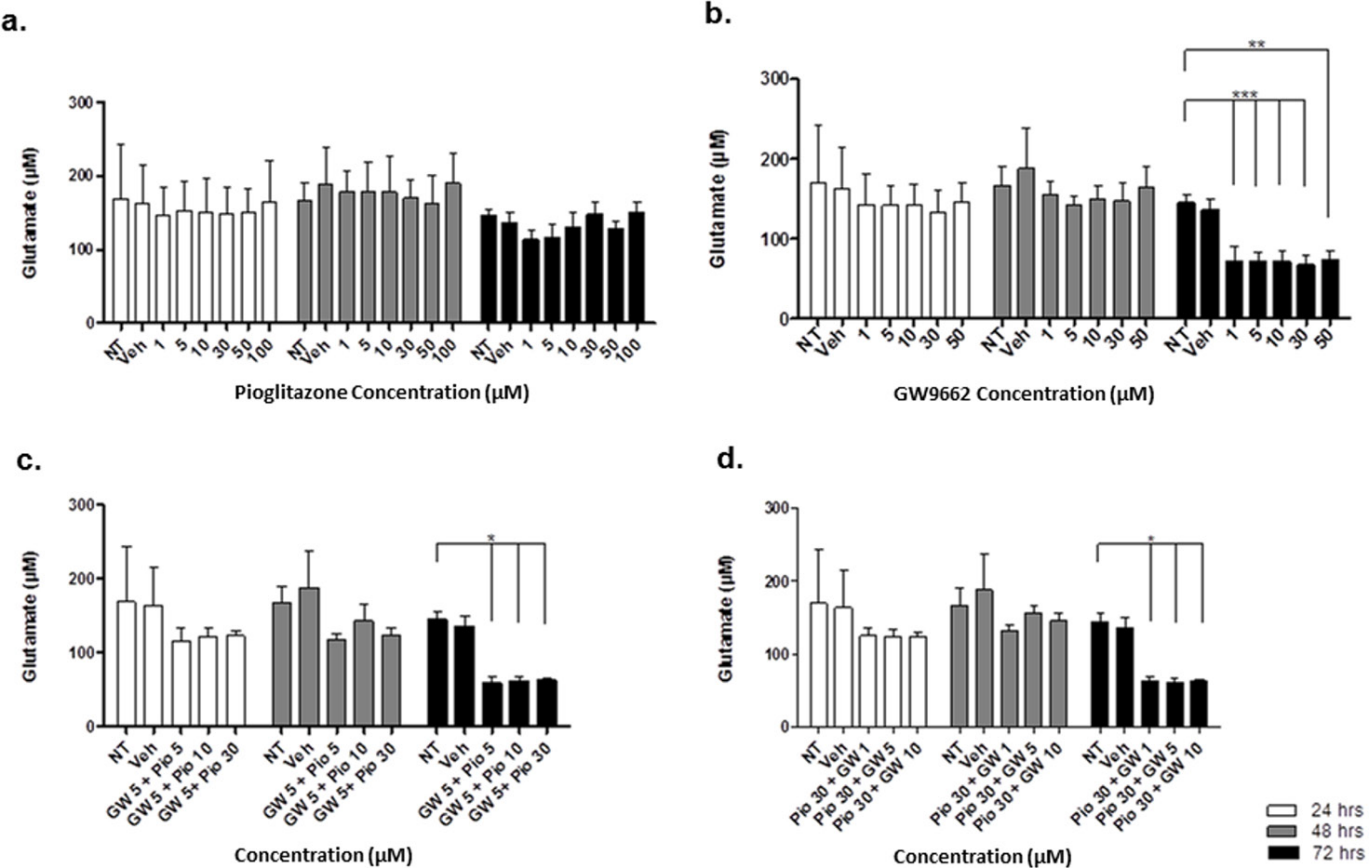
Extracellular glutamate levels measured with HPLC in GSC #035 cells incubated with varying concentrations of pioglitazone (a) GW9662 (b) GW9662 + pioglitazone (c & d) for 24, 48 and 72 hours (*n* = 6) A significant reduction in glutamate release was elicited at a concentration of ≥ 1 μM of GW9662 at 72 hours (b–d) There was no significant difference in glutamate release administration of pioglitazone alone (a). Bars show mean with SEM and asterisks indicate statistical significance where *p = 0.01 to 0.05, **p = 0.001 to 0.01, ***p < 0.001, and ****p < 0.0001. ANOVA with Bonferroni Post hoc test, *n* = 6. NT: non-treated, Veh: Vehicle control, Pio: Pioglitazone, GW: GW9662.

In order to elucidate whether glutamate uptake in glioma cells is increased by any potential reduction in glutamate transporters, we measured glutamate levels in the glioma culture media. As the glioma cell lines U87MG and U251MG were cultured in media containing 5% FCS, a high glutamate background was expected as has been previously reported. [30] We determined the background glutamate concentration in culture medium was 34.16 μM ± 12.08 (data not shown). In U87MG cells we observed a significant dose-dependent reduction in extracellular glutamate levels with increasing concentrations of pioglitazone ≥ 30 μM at 72 hours (Figure 7(a)). A similar observation was made with U251MG (Figure 8(a)). This correlated with the change in protein expression, where increased EAAT2 expression was highest at pioglitazone concentrations of 30 μM and 10 μM in U87MG and U251MG respectively (Figure 2(a) & (b)). These results suggest that pioglitazone-induced upregulation of EAAT2 leads to increased uptake of extracellular glutamate. These results were not corroborated in GSC #35, where no significant change in extracellular glutamate was elucidated (Figure 9(a)). Although GW9662 was associated with a significant reduction in glutamate in U87MG and U251MG cells at ≥ 30 μM and 50 μM respectively (Figure 7(b) & 8(b)), these concentrations were associated with a significant reduction in cell viability (Figure 4(c) & 4(d)).

### Pioglitazone mediated reduction in extracellular glutamate is PPARγ dependent

To confirm whether EAAT2 modulation in glioma cells using pioglitazone was PPARγ dependent, the U87MG and U251MG glioma cell lines were treated with GW9662 alone and co-administration of pioglitazone and GW9662. We demonstrated that the lowest effective concentration of pioglitazone of 30 μM in both cell lines is inhibited by concentrations of GW9662 as low as 1 μM (Figures 7(c) & (d) & 8(c) & (d)). Therefore, the capacity for pioglitazone to reduce extracellular glutamate is highly dependent on PPARγ in cell lines. In GSC #35, the converse is true, whereby GW9662 is associated with significantly reduced extracellular glutamate levels at 72 hours exposure alone (Figure 9(b)) or in combination with pioglitazone (Figure 9(c)–(d)).

### Pioglitazone alters GBM cell line morphology and reduces glioma sphere formation

**Figure 10:**
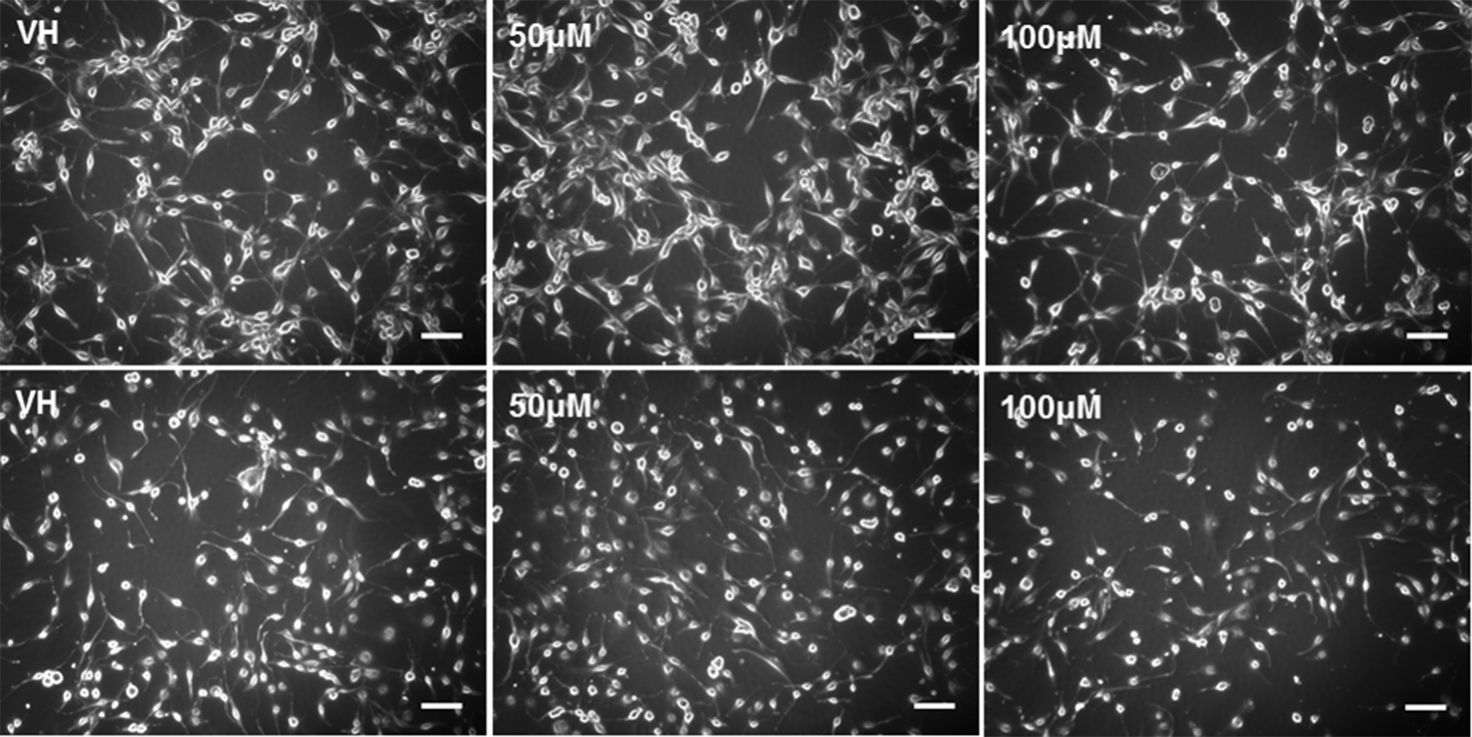
Top row: U87MG treated with increasing doses of pioglitazone for 48 hours Cellularity and formation of spreading astrocytic process formation were reduced with 100 M pioglitazone compared with vehicle control (VH). Cells were smaller and numerous detached cells were present but quantity and length of astrocytic processes were unchanged at 10X objective. Bottom row: U251 cells cultured for 48 hours treated with increasing concentrations of pioglitazone. Cellularity and formation of astrocytic processes decreased at concentrations above 10 μM at 10X objective. Scale bars = 100 μm.

**Figure 11:**
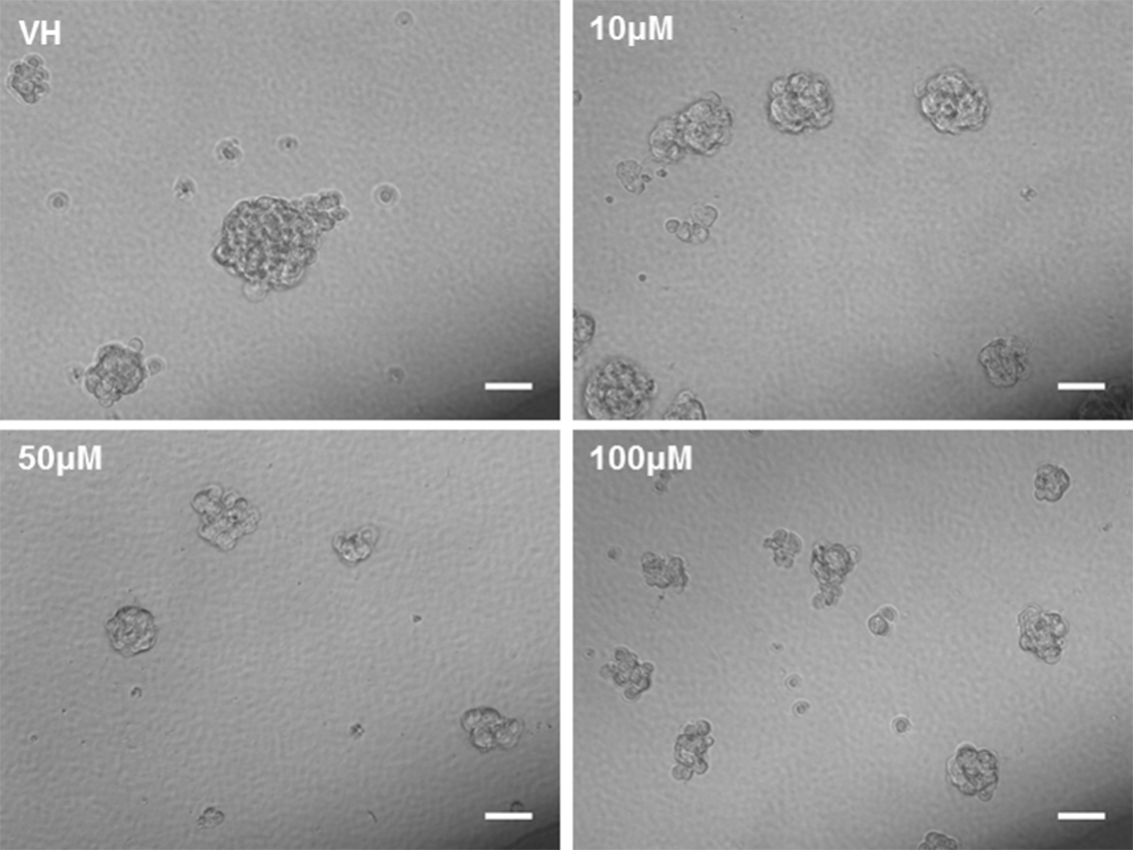
Phase contrast photos at 20X objective of glioma stem cell line #035 incubated with vehicle control (VH) and increasing concentrations of pioglitazone for 48 hours Pioglitazone reduces the quantitative formation and size of neurosphere formation. Scale bars = 50 μM.

**Figure 12:**
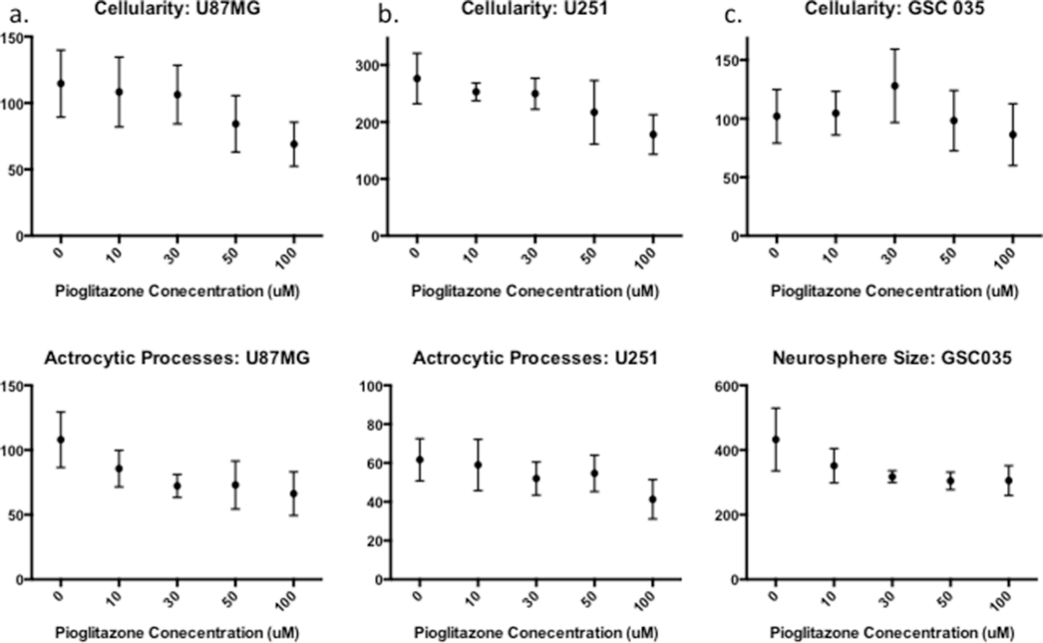
Quantification of photomicrograph cellularity and morphological change Increasing concentrations of Pioglitazone 30 μM or above significantly reduces cellularity and astrocytic process formation in U87MG **(a)** but not in U251MG **(b)** Glioma stem cell neurosphere formation (cellularity) reduces significantly with a concentration of 100 μM of Pioglitazone **(c)**, however, the average area of neurospheres reduces but does not change significantly in these conditions. For all experiments *n* = 3.

As it has previously been established that PPARγ agonists have cytotoxic and cytostatic activity, we sought to determine if pioglitazone can alter glioma cellular morphology. [19, 31] In U87MG glioma cells, we observed that cellular morphology was transformed at a pioglitazone concentration of 100 μM with a reduction in cellularity, coupled with a compromised ability to form astrocytic processes (Figure 10). A similar observation was made in U251MG glioma cells at a pioglitazone concentration of 100 μM (Figure 10). Glioma sphere formation, as assessed in the GSC #35 line, was reduced with the pioglitazone treatment but sphere size was not significantly reduced (Figure 11). Quantification of these observations correlates well with viability results (Figure 12).

### Pioglitazone may alter Akt and GSK-3β activity

**Figure 13:**
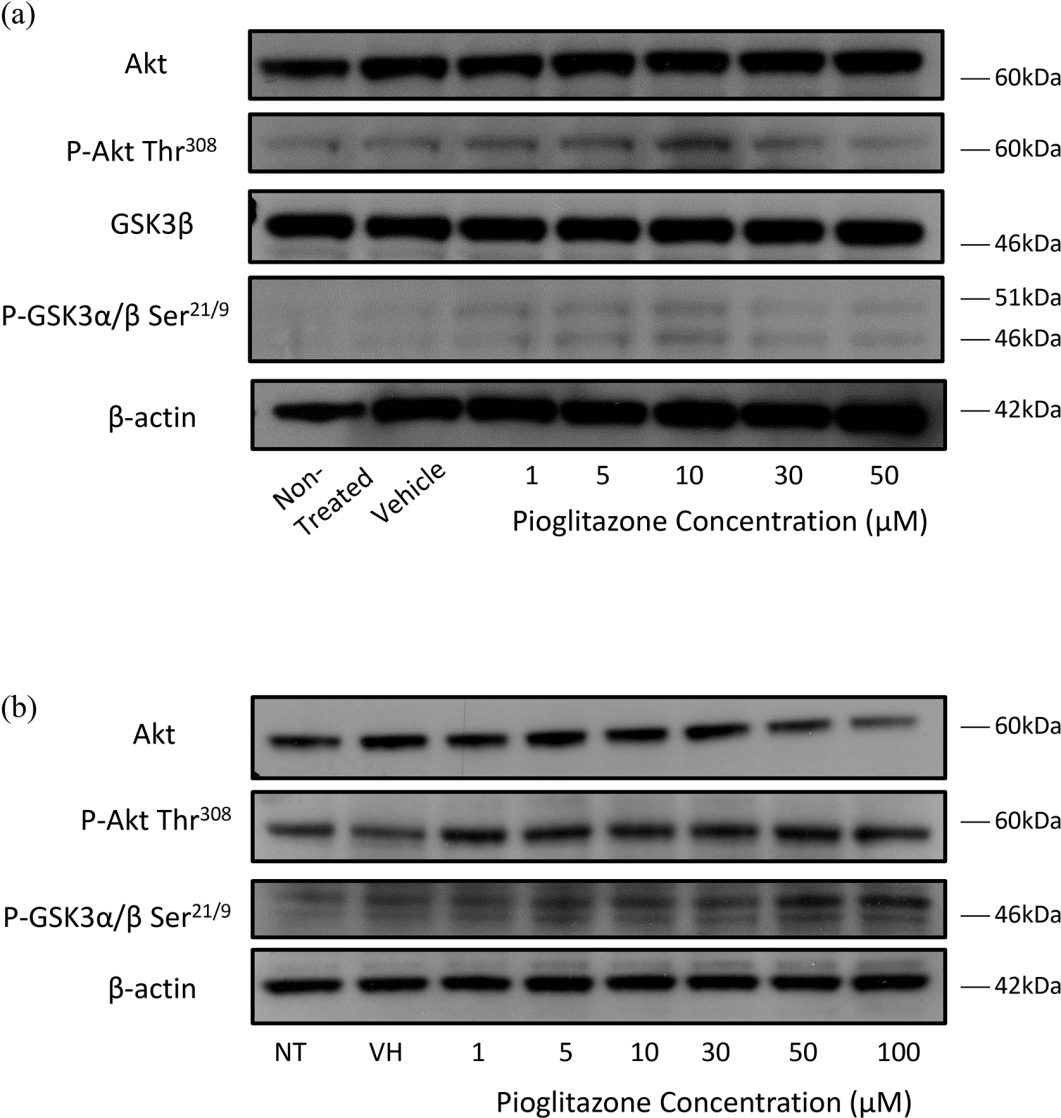
(a) Representative blots showing Akt, p-Akt, GSK-3β and p-GSK-3α/β and β-actin expression in U87 glioma cells treated with increasing concentrations of pioglitazone for 72 hours **(b)** Representative blots showing Akt, p-Akt, p-GSK-3β and β-actin expression in U251MG glioma cells treated with increasing concentrations of pioglitazone for 48 hours.

In order to understand how pioglitazone may reduce cell viability in glioblastoma cell lines, we sought to measure the expression of pertinent oncogenic signalling cascades. Lee and colleagues have previously demonstrated that ciglitazone treatment results in reduced phosphorylation of Akt (Thr^308^), which is not altered by the presence of the PPARγ inhibitor, GW9662. [18] We observed that in U87MG cells treated with pioglitazone there was increased expression of both phosphorylated Akt and phosphorylated GSK-3β with no change in the levels of total Akt or GSK-3β (Figure 13(a)). In contrast, when treating U251MG cells with pioglitazone, we did not detect a significant change in the phosphorylation status of GSK-3β, although total Akt protein levels were marginally reduced with 100 μM pioglitazone (Figure 13(b)). This suggests that pioglitazone operates through a different mechanism of apoptotic cell death in glioma cells compared to ciglitazone.

## DISCUSSION

Our study provides evidence that the PPARγ agonist pioglitazone increases the functional expression of the glutamate transporter EAAT2 in glioma cells. This represents a potential novel mechanism for treatment of glioma associated seizures, whereby excess extracellular glutamate is transported back into glioma cells and also by normal astrocytes at the peritumoural edge. This is a potentially therapeutic approach by preventing glutamate mediated seizures and excitotoxic damage. In light of the findings by Romera and colleagues that PPARγ agonists increase astrocytic EAAT2 and subsequently reduce extracellular glutamate, which we show that PPARγ-mediated EAAT2 modulation is also applicable to glioma cells. [32] We did not carry out a glutamate uptake assay, which would be important to confirm true functional upregulation. [6] However, cell viability was not significantly reduced at the effective concentration of 30 μM of pioglitazone in U251MG glioma cells reported here, providing a surrogate for EAAT2 function. Whether pioglitazone also affects glutamate release, glutamate uptake or both requires further investigation.

De Groot and colleagues previously demonstrated that overexpression of EAAT2 in glioma cell lines, including U87MG and U251MG glioma cells, resulted in a reduced ability to form tumours and also caused *in vivo* and *in vitro* apoptosis. [29] Pioglitazone induced EAAT2 upregulation may play a role in reducing glioma cell viability via multiple mechanisms, particularly through the activation of caspase-3, which has been shown in both overexpression studies and cellular apoptosis assays. [29, 33]

Deactivation of oncogenic mediators such as Akt and β-catenin has been implicated as possible PPARγ independent mechanisms for PPARγ agonists. [18, 19] We observed a reduction in total Akt protein expression levels in U251MG glioma cells following treatment with 50 and 100 μM of pioglitazone. This was in contrast to the increase in Akt protein expression detected in U87MG cells, where Akt phosphorylation at the threonine 473 (thr308) residue was unchanged in U251MG cells but increased in U87MG cells (Figure 13). Lee and colleagues observed that a concentration of 30 μM pioglitazone effectively reduced total Akt activity and phosphorylation at threonine 308 (thr308) residue, but not ser473 in T98G cells after 48 hours. [18] The increased phosphorylation of Akt thr^308^ was not seen to significantly reduce cell viability when cells were incubated with pioglitazone. Reduction in Akt activity is desirable to counteract mutations in RTK/PTEN/PI3K in gliomas that lead to increased Akt activity downstream, resulting in uncontrolled proliferation.

We discovered that GSK-3β expression levels were decreased with a corresponding increase in GSK-3α and GSK-3β phosphorylation on ser^21^ and ser^9^ residues, respectively, in U87MG cells treated with pioglitazone (Figure 13) but not in U251MG after 72 hours (data not shown), indicating inactivation of GSK-3β. However, we did not observe a correlation between Akt mediated GSK-3β inactivating phosphorylation as reported by Atkins and colleagues. [34] This implies that PPARγ agonists may inactivate GSK-3β through other upstream mediators alongside or independently through direct inhibition. [25] The more noticeable reduction in astrocytic processes formation in U251MG over U87MG cells may not implicate GSK-3β inhibition as a downstream target of PPARγ agonists, which has been previously shown with lithium chloride of U87MG cells. [35] We also did not detect an association of pioglitazone mediated EAAT2 expression or the activation state of GSK-3β. In addition GSK3 inhibition by pioglitazone may also prevent the formation of neurospheres (Figures 11 & 12), corroborating previous findings by Korur and colleagues. [28] Wan and colleagues previously demonstrated that pioglitazone significantly reduces viability of U87MG and U251MG cells at concentrations of 100 μM and 200 μM beyond 72 hours. [19] Our results demonstrate that pioglitazone may be more potent in this role as we observed a significant reduction in cell viability at concentrations of ≥ 30 μM in U87MG and 100 μM in U251MG as early as 24 hours.

In order to determine whether the observed effects on viability and EAAT2 modulation relied on PPARγ activation, the inhibitor GW9662 was used as previously described in studies utilising breast tumours and glioma cell lines. [18, 36] In our current study, GW9662 alone reduced cell viability of U87MG and U251MG cells at concentrations of at least 10 and 30 μM, respectively. A potential anti-neoplastic pathway for GW9662 is through the reduced expression of Fatty Acid Binding Protein 7 (FABP7), which has been implicated as a target gene for PPAR. [37] Authors of this study demonstrated a reduction in cellular migration after siRNA knockdown of FABP7 and the PPAR antagonist treatment of GSCs with and without irradiation. This potentially contradicts previous findings that PPARγ agonists induce apoptosis in GSCs, however the effect of PPARγ agonists on FABP7 has yet to be thoroughly investigated. [38] Furthermore, these two studies differ in the use of primary and enriched long-term GSC lines, which may explain the differences to previous studies.

We observed no change in EAAT2 expression in glioma stem cell line #035 with pioglitazone exposure, which may be in keeping with a lineage specific GSC as shown by Pollard and Colleagues. [39] Extracellular glutamate levels were not raised in the culture medium from GSC #35 and pioglitazone treatment did not augment this (Figure 9). Interestingly, Gilley and colleagues found that neural stem cells derived from the hippocampal dentate gyrus exhibited raised EAAT2 expression in hypoxic-ischaemic and traumatic brain injury rodent models. [40] Furthermore, these authors showed that siRNA knockdown of EAAT1 and EAAT2 resulted in an increase in neurosphere formation and proliferation, whereas overexpression had the converse effect. This mirrors our findings, which showed that GSC #035 does not express EAAT1 or 2 and there was reduced formation of glioma stem-like neurospheres with increasing concentrations of pioglitazone. This raises important questions about how GSCs survive in a microenvironment that is rich in glutamate. It could be the case that this alters their proliferative ability, which may ultimately be involved in selecting a subpopulation of GSCs. Our data from one GSC line suggests that such cells can release low levels of glutamate around 10 μM (data not shown).

The morphological effects of pioglitazone on glioma cells has not been previously reported. Herein we have shown that pioglitazone treatment alters the morphology of U87MG, U251MG glioma cells and a primary GSC line in a concentration dependent manner. However, the observed changes in cell morphology did not correlate with cell viability. U87MG morphology notably changed in the presence of 100 μM pioglitazone but cell viability was significantly reduced at concentrations of 30 μM and above. GW9662, an inhibitor of PPARγ demonstrated opposing effects in combination with a cytostatic dose of pioglitazone, which may be due to PPARγ independent effects of the two drugs working synergistically as reported elsewhere. [13].

Bright and colleagues previously demonstrated the efficacy of the more potent PPARγ agonist ciglitazone on CD133^+^ U87MG and T98G neurospheres. [38] A cytotoxicity assay on our GSC line #35 with increasing concentrations of pioglitazone did not corroborate these findings (Figure 6(a)–6(h)). However, our GSC line was not de-differentiated from established cell lines, rather isolated from a primary resection. Furthermore, there may be unexplored PPARγ-independent mechanistic differences between pioglitazone and ciglitazone that are not accounted by the class effect of these drugs.

Owing to the established association between glutamate and tumour associated epilepsy, there may also exist a novel role for PPARγ agonists. [9, 10] Notably, we chose the U251MG cell line since Buckingham and colleagues previously utilised this in establishing a tumour associated epilepsy model in rodents. [10] Although pioglitazone is associated with a minor risk of bladder cancer, it is otherwise a relatively safe drug that could be considered for clinical trials. [41] Alternatively, 293 agents that upregulate EAAT2 protein expression in primary astrocytes have been identified with high throughput screening, providing a new family of candidate drugs that are available to be tested for this treatment strategy. [42]

Currently, the only promising drug candidate for tumour associated epilepsy has been sulfasalazine. [10] Extensive clinical trials have yet to follow, but a previous clinical trial suggests potential challenges with this agent. [43] Sattler and colleagues have recently demonstrated that thiamphenicol, through its upregulation of EAAT2 in peritumoural tissue, may be similarly beneficial. [44] PPARγ agonists may constitute an additional agent that activates glutamate transport within tumour cells and peritumourally. These agents could be considered for adjunct treatment in current regimens and in combination therapy for dual mechanistic efficacy in sub therapeutic doses to avoid associated adverse effects.

## MATERIALS AND METHODS

### Reagents

Dulbecco's Modified Eagle's Medium (Invitrogen, Camarillio, CA, USA) heat inactivated foetal calf serum (Bovogen, Victoria, Australia) and penicillin/streptomycin (Invitrogen, Camarillo, CA, USA) were source listed. Pioglitazone hydrochloride was obtained from Sigma Aldrich (St. Louis, MO, USA) and GW9662 from Santa Cruz Biotechnology Inc. (Santa Cruz, CA, USA). Primary antibodies for EAAT 2 (rabbit polyclonal IgG, sc-15317) and PPARγ (mouse monoclonal IgG, sc-7273) were purchased from Santa Cruz Biotechnology Inc. (Santa Cruz, CA, USA) and EAAT1/GLAST1 (rabbit polyclonal, cat. #42–8100) from Invitrogen (Camarillo, CA, USA). Primary antibodies for Akt (polyclonal rabbit, cat. #9272), phosphorylated-Akt (ser 473) (polyclonal rabbit, cat. #9271), glycogen synthase kinase 3 beta (GSK3β) (polyclonal rabbit, cat. #9315) and phosphorylated-GSK3α/β (Serine 21/9) (cat. #9331) were purchased from Cell Signalling Technology Inc. (Danvers, MA, USA). β-actin primary antibody (mouse monoclonal, cat. #A2228) was purchased from Sigma Aldrich (St. Louis, MO, USA). Goat anti-rabbit antibody conjugated with horse raddish peroxidase (cat. #170–6515) and goat anti-mouse antibody conjugated with horse raddish peroxidase (cat. #170–6516) was purchased from Bio-Rad (Hercules, CA USA). Cell Titer Glo Cell Luminescent Viability Assay was purchased from Promega (Madison, WI, USA). Protein estimation of whole cell lysates was carried out using the BCA Protein Assay Reagent (bicinchoninic acid) from Thermo Scientific Rockford, IL, USA). Western Blot reagents, apparatus, and 4–12% Bis-Tris pre-cast gels were purchased from Invitrogen (Camarillo, CA, USA).

### Cell culture

Glioma cell lines U87MG and U251MG cells were obtained from the American Type Culture Collection, Manassas, VA, USA were maintained in DMEM supplemented with 5% foetal bovine serum and penicillin/streptomycin at 37°C and 10% carbon dioxide.

A human glioblastoma stem cell line (GSC #35) was previously generated in our laboratory from a brain tumour tissue specimen (Melbourne Health Research Ethics 2009.016). Neurospheres produced from GSC #35 maintained in DMEM F12 supplemented with epidermal growth factor (1:1000), basic fibroblast growth factor (1:1000), B27 (1:50) and penicillin/streptomycin at 37°C and 10% CO_2_ using ultra low adhesion 6 well plates, as previously described by Singec et al. [45].

### Preparation of rodent brain samples

Non-epileptic control Wistar rats were used for control samples and were obtained from our own breeding colony (Biological Resource Facility, RMH Academic Centre). All procedures were approved by the University of Melbourne Animal Ethics Committee (AEC #1111944) and performed in accordance with the guidelines published by the Australian NHMRC for use of animals in research.

Seven day old Wistar rat pups were culled and samples of cortex and thalamus were stored on dry ice. Whole cell lysates were prepared in erythrocyte lysis buffer (ELB) (500 mM NaCl, 100 mM HEPES, and 10 mM EDTA, 0.2% Triton X100, 20 mM NaF, 2 mM NaV, 2 μg/mL aprotonin). The whole cell lysates were clarified by centrifugation at 13,000 rpm at 4°C for 10 minutes.

### Photomicroscopy and quantification of cell morphology

ImageJ (ver. 1. 48, NIH, Bethesda, Maryland) was utilised to measure cellularity, formation of astrocytic processes, and neurosphere formation and size. Cellularity was measured by converting images to a binary format and using processing tools to demarcate cells for automated counting. Average area of neurospheres was used to estimate the size of neurosphere formation. Astrocytic processes were measured manually using binary processes in ImageJ.

### Drug treatment of cell lines

Pioglitazone hydrochloride (Sigma Aldrich, St. Louis, MO, USA) and GW9662 (Santa Cruz, CA, USA) were dissolved in dimethyl sulfoxide (DMSO) and stored at −20°C until use. Glioma cells were treated at a number of drug concentrations and times as indicated in the results section.

### Cell viability assay

1 × 10^4^ glioma cells were seeded in each well of a 96 well plate and at various time points (24, 48 and 72 hours), the culture medium was aspirated and 50 μL of Cell Titre Glo (Promega, Madison, WI, USA) viability solution was added. The plate was incubated at 4°C with gentle rocking for 20 minutes and 40 μL of the viability solution from each well was then transferred to an opaque 96 well plate and luminescence measured with using a GloMax^®^ Microplate Luminometer (Madison, WI, USA). Viability was compared with untreated cells at 24, 48 and 72 hours).

### Western blotting

Adherent cells were washed with PBS and lysed with ELB over ice. Proteins were then separated by SDS-PAGE on 4–12% Bis-Tris gels (Invitrogen), transferred onto polyvinyldene fluoride membrane and probed with the indicated primary antibodies. The signal was visualised using the ECL chemilluminescence detection kit (GE Healthcare, Rydelmere, N.S.W., Australia) following incubation with appropriate secondary antibodies.

### High performance liquid chromatography

Glutamate analysis in cell culture media was performed using the isocratic HPLC method which utilises naphthalene-2, 3-dicarboxyaldehyde derivatisation (NDA) and subsequent fluorescence detection as previously described. [46]

### Statistics

All statistical analysis and graph generation was performed using the software package GraphPad Prism 5.0, La Jolla, CA, USA. The unpaired Student's *t*-test was used to compare groups and statistical significance was considered to be *p* ≤ 0.05.

## CONCLUSIONS

The PPARγ agonist pioglitazone increases functional EAAT2 expression and reduces extracellular glutamate levels in both U87MG and U251MG glioma cells. Pioglitazone alters the cell morphology and reduces cell viability of the glioma cell lines, U87MG and U251MG. Pioglitazone and other PPARγ agonists may offer a novel treatment paradigm for the treatment of TAE through the promotion of extracellular glutamate clearance at both the glioma cell level and surrounding astrocytes. Further work is required to definitively elucidate the mechanisms of this therapeutic strategy.
